# Fusion with stem cell makes the hepatocellular carcinoma cells similar to liver tumor-initiating cells

**DOI:** 10.1186/s12885-016-2094-7

**Published:** 2016-02-04

**Authors:** Ran Wang, Shuxun Chen, Changxian Li, Kevin Tak Pan Ng, Chi-wing Kong, Jinping Cheng, Shuk Han Cheng, Ronald A. Li, Chung Mau Lo, Kwan Man, Dong Sun

**Affiliations:** Department of Mechanical and Biomedical Engineering, City University of Hong Kong, Hong Kong, China; Department of Surgery, Li Ka-Shing Faculty of Medicine, University of Hong Kong, Hong Kong, China; Stem Cell and Regenerative Medicine Consortium, and Departments of Medicine and Physiology, Li Ka-Shing Faculty of Medicine, University of Hong Kong, Hong Kong, China; Environmental Science Program, School of Science, Hong Kong University of Science and Technology, Hong Kong, China; Department of Biomedical Science, City University of Hong Kong, Hong Kong, China

**Keywords:** Cell fusion, Hepatocellular carcinoma, Stem cell, Tumor-initiating cell

## Abstract

**Background:**

Cell fusion is a fast and highly efficient technique for cells to acquire new properties. The fusion of somatic cells with stem cells can reprogram somatic cells to a pluripotent state. Our research on the fusion of stem cells and cancer cells demonstrates that the fused cells can exhibit stemness and cancer cell-like characteristics. Thus, tumor-initiating cell-like cells are generated.

**Methods:**

We employed laser-induced single-cell fusion technique to fuse the hepatocellular carcinoma cells and human embryonic stem cells (hESC). Real-time RT-PCR, flow cytometry and in vivo tumorigenicity assay were adopted to identify the gene expression difference.

**Results:**

We successfully produced a fused cell line that coalesces the gene expression information of hepatocellular carcinoma cells and stem cells. Experimental results showed that the fused cells expressed cancer and stemness markers as well as exhibited increased resistance to drug treatment and enhanced tumorigenesis.

**Conclusions:**

Fusion with stem cells transforms liver cancer cells into tumor initiating-like cells. Results indicate that fusion between cancer cell and stem cell may generate tumor initiating-like cells.

## Background

Cell fusion is a cellular process of combining two or multiple cells to form a single entity. Today, fusion with stem cells is used to induce somatic cell nuclei reprogramming and study the cell reprogramming mechanism [1]. Cell fusion is also a fast and efficient approach to induce pluripotent stem cells. The ability of somatic cells to acquire homologous characteristics and be converted to pluripotent cells with minimal modification have been demonstrated through the fusion of stem cells [2–4].

Hepatocellular carcinoma (HCC) is a malignant tumor with high mortality rate. New therapeutic strategies against liver cancer are widely discussed in research and clinical medicine. Stem cell research is a potential approach to liver cancer treatment [5]. Examples of these approaches include the injection of stem cell for damage repair [6], liver regeneration [7], and induced differentiation of hepatocyte cells [8].

This paper presents a study on engineered fusion of stem cells and cancer cells to explore mechanism of cancer stem cell that may produce potential influence to stem cell therapy for liver cancer treatment. Through laser-induced fusion between a single hepatocellular carcinoma cell (HepG2) and a human embryonic stem cell (hESC), we determined that the fused cell acquired stemness and cancer-like characteristics. The fused cells exhibited increased tumorigenicity and resistance to drug treatment. The gene expression analysis indicated that the fused cells were highly similar to tumor stem cells (TSCs) or tumor-initiating cells (TICs). TICs are important in tumorigenesis, aggravation, metastasis, and recurrence [9, 10]; liver TICs and their markers were identified [11–13]. Based on the results of gene expression analysis and quantity PCR detection, we found that some liver TIC markers such as CD133, CD44, and ALDH1 exhibited significantly high expression in the fused cells. However, this condition was not observed in HepG2 cells. The in vivo tumorigenicity assay and drug resistance assay also demonstrated that the fusion of cancer cells with stem cells resulted in cancer cells that were highly similar to cancer stem cells. These results suggested the possibility of incurring risks when some stem cell therapies are used for cancer treatment.

## Methods

### Cell culture

The hESC line HES2 (NIH code: ES02) from ES Cell International (passage 60 to 84) was cultured on a commercially available, serum-free, and feeder-independent system (mTeSR1, Stemcell Technologies) and supplemented with BD Matrigel hESC-qualified Matrix (BD Catalog #354277), as suggested by the manufacturer. Genetically labeled *Oct4*-GFP^+^ hESCs were generated by transduction (MOI = 5) with lentiviral vectors carrying a cassette of *Oct4* promoter-driven GFP.

HepG2 cells were maintained in DMEM (Gibco, Catalog #11965-092), supplemented with 10 % fetal bovine serum (FBS, Gibco, Catalog #10270-106), 100 U/mL penicillin, and 100 U/mL streptomycin (Invitrogen, Catalog #15240-062) at 37 °C in a humidified atmosphere of 5 % CO^2^.

### Cell fusion

Before the fusion experiment, the single cells were obtained with the enzymatic treatment for passaging. After proper neutralization, the single-cell suspension in the culture medium was stored temporarily at 4 °C. To distinguish hESCs from HepG2, hESCs were labeled with Oct-GFP and HepG2 cells were stained with red mitochondrion selective probe. For mitochondria staining, the HepG2 single cells were incubated in a 40 nM MitoTracker probe (Life Technology) for 15 min and washed three times. The stained cells were re-suspended in DMEM with 10 % FBS.

**Fig. 1:**
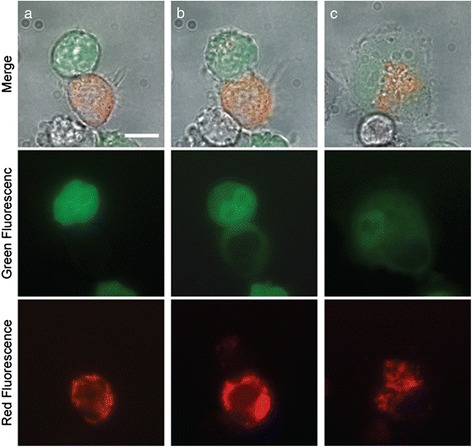
Laser-induced cell fusion of hESC and HepG2. **a** GFP-labeled hESC (green) and mitochondria-labeled HepG2 (red) forming a cell pair before laser cutting. **b** GFP transfer from the green cell to the red cell after laser cutting. **c** The fused cell attracted to the coverglass surface and exhibited adherent cell morphology about 25 min after laser cutting. (Scale bar = 20μm)

The fusion experiment was performed, as described in the literature [14]. One GFP labeled hESC and one mitochondria-stained HepG2 cell were trapped and manipulated using optical tweezers to form a cell pair. Laser scissors functioned at 10 Hz, and each pulse had a duration of 1 ns to cut the cell membrane at the point of contact between the two cells. Successful fusion was verified by observing the transfer of cytoplasmic GFP from the hESC to the HepG2 cell (as shown in Fig. 1). The fused cells attached to the cover glass bottom and survived.

### Cell isolation and colony formation

A total of 24 h after fusion, the fused cells were collected following the trypsin-EDTA treatment and diluted in a low-density single-cell suspension (6 cells/mL to 10 cells/mL). In each well of a 96-well plate, 100 μL of the cell suspension was seeded. After 24 h of incubation, the plate was examined under a microscope, and the wells containing single cells were selected. Cells were fed with new DMEM medium every 2 days. After one week of culturing, colonies formed in the marked wells, and these colonies, which exhibited different morphologies than that of HepG2, were gradually replanted to 35 mm dishes for further characterization.

### Gene sequencing and differential gene expression analysis

For each sample, the total RNA was sent to the BGI Company for RNA-Seq (Quantification) sequencing and screening of differentially expressed genes (DEGs).

### Flow cytometry

We performed FACS tests on HepG2, fused cells and hESCs, respectively. Cells were detached from culture dishes and suspended in 100 μl of buffer (Miltenyi Biotec) and human serum (1:1) for 15 min at 4°C. Cells were then incubated for 45 min with a primary antibody for AFP (Santa Cruz Biotechnology, Cat No. sc-8399) and CD133-PE (Miltenyi Biotec, Cat No. 130-098-829). Cells were washed with FACS buffer, and incubated for 30 min at 4°C with a secondary antibody (Santa Cruz Biotechnology, Cat No. sc-2856). Cell labeling was detected using FACSVerse^TM^ (BD Pharmingen). Flow cytometry results were analyzed by using BD FACSuite software.

### Quantitative RT-PCR

Total RNA was extracted from the cells with Trizol reagent (Invitrogen) using the method provided by the manufacturer. Reverse transcribed cDNA was produced with iScript^TM^ cDNA synthesis kit (Bio-Rad, Cat No170-8890). Real-time quantitative PCR amplification was performed with SsoAdvanced SYBR Green supermix kit (Bio-Rad, Cat No. 1725260) in CFX96 Real-time System (Bio-Rad, USA). The specific primers used in the analyses are listed in Table 1. The CD133 and CD44 primers were described in [15] and [16]. Additional primer sequences were obtained from the Primer Bank [17].

**Table 1:** Specific primers used in qPCR

Gene	Forward primer (5’-3’)	Reverse Primer (5’-3’)
AFP	AGACTGAAAACCCTCTTGAATGC	GTCCTCACTGAGTTGGCAACA
CD133	ACATGAAAAG ACCTGGGGG	GATCTGGTGTCCCAGCATG
CD44	TCCCAGACGAAGACAGTCCCTGGAT	CACTGGGGTGGAATGTGTCTTGGTC
ALDH1A1	CTGCTGGCGACAATGGAGT	CTGCTGGCGACAATGGAGT
ABCB1	GGGAGCTTAACACCCGACTTA	GCCAAAATCACAAGGGTTAGCTT
EpCAM	AATCGTCAATGCCAGTGTACTT	TCTCATCGCAGTCAGGATCATAA
Bcl-2	GACTGAATCGGAGATGGAGACC	GCAGTTCAAACTCGTCGCCT
β-actin	CATCCTCACCCTGAAGTACCC	AGCCTGGATAGCAACGTACATG

### Drug resistance

The fused cells and HepG2 were first incubated in a 6-well plate for 15 h. After this step, 0.1, 0.2, and 0.5 μm DOX were added to the culture medium with three parallel samples for every group. The cells were incubated for an additional 48 h. Cell viability was determined through the trypan blue exclusion test.

### In vivo tumorigenicity assay

HepG2 cells and the fused cells (5×10^4^, 1×10^5^, and 1×10^6^ cells in 100 ul DMEM) were subcutaneously injected into the right scapula of each nude mouse (BALB/c nu/nu, 4 to 6 weeks old). Four nude mice were prepared for each group. Tumor growth in the nude mice was measured every 7 days for 6 weeks. The nude mice were sacrificed at week 6 or 7. The tumors were fixed with formaldehyde solution and stained with hematoxylin-eosin (H&E).

### Animal care and ethics statement

Male athymic nude mice (BALB/c nu/nu, 4 to 6 weeks old) were used in this study. These animals were housed in pathogen-free conditions and provided with food and water at the facility of LKS Faculty of Medicine, University of Hong Kong (HKU). The Committee on the Use of Live Animals in Teaching and Research, HKU approved the protocol.

### Statistical analysis

Data on RT-PCR results and drug resistance were expressed as mean ± SD. Data from in vivo tumor experiment was expressed as mean ± s.e.m. Statistical analysis was performed by adopting the two-tailed Student’s *t*-test. Differences with p ≤ 0.05 were considered significant.

## Results

### Gene expression of the fused cells is different from HepG2 cells

**Fig. 2:**
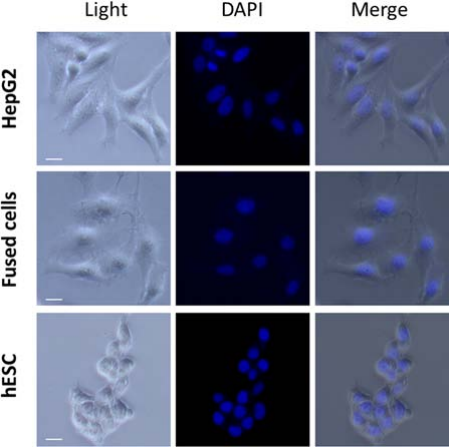
Morphology of HepG2, fused cells and hESc. Morphology of the fused cells differs from that of HepG2 cells and hEScs. (Scale bar = 20μm)

**Fig. 3:**
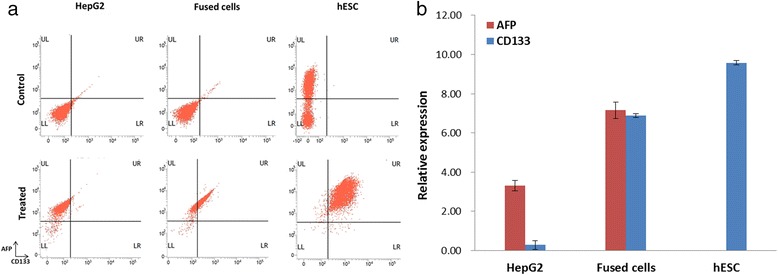
Representative gene marker expression in HepG2 and fused cells. **a** Flow cytometer analysis for the expression of marker protein AFP and CD133 in HepG2, fused cells, and hESCs. Cells in the control group were not antibody incubated while cells in the treated group were incubated with AFP and CD133 antibody. As hESCs were labeled with GFP, hESCs from both the control and treated groups were green positive population. **b** Gene marker expression analysis shows that both AFP and CD133 were expressed in the fused cells, indicating that both HepG2 and hEScs affected the gene expression in the fused cells

As shown in Fig. 2, the isolated fused cells did not exhibit a conspicuous spindle shape, which was different from the morphology of HepG2 cells and hESC. The marker protein and gene expression of the fused cells and their parent cells were also detected, and the results showed that both HepG2 and hESCs affected the fused cells (see Fig. 3). To identify the functional difference between the fused cell and the HepG2 cells, we conducted pairwise comparisons to examine the significantly different molecular function, biological process, and cellular component (Gene Ontology [GO]). As indicated in Table 2, the immune response, immune system process, and regulation of apoptosis of the differential expression genes improved. Similar to CD133, CD44 exhibited a different expression in the fused cells and HepG2 cells in the analysis. We conducted qPCR tests for the related genes. The results showed that several liver tumor-initiating cell markers, including AFP, CD133, CD44, and EpCAM [18], were unregulated in the fused cells compared with those of HepG2 cells (Figs. 3b and 7). CD133 is an important stemness biomarker in normal stem cells and TICs, which can help maintain tumorigenic property and affect the tumor growth of the cells [19]. CD44 invariant regulates the redox status, which affects tumor initiation, tumorigenesis, and metastasis [20]. CD133 and CD44 are rarely expressed in normal cancer cells. EpCAM is a key protein that activates the Wnt signaling pathway, which is related to tumorigenesis, invasion, and resistance to drug treatments. The high expression of EpCAM enhances the chemoresistance of cells [21–23].

**Table 2:** Significantly enriched GO terms in DEGs

Gene ontology term	Cluster frequency	Genome frequency of use	Corrected *p*-value
Immune response	10.2 %	4.9 %	2.74e-07
Immune system process	12.4 %	6.8 %	3.77e-06
Response to virus	2.6 %	0.8 %	0.00138
Regulation of apoptosis	7.8 %	4.3 %	0.00508
Regulation of programmed cell death	7.8 %	4.4 %	0.00840
Regulation of cell death	7.8 %	4.4 %	0.00888

### Fused cells exhibited increased drug resistance

**Fig. 4:**
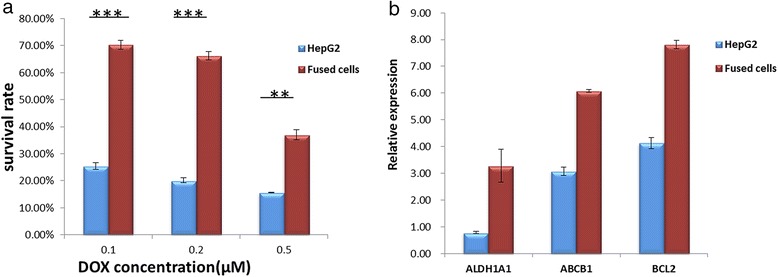
Drug resistance and related gene expression analysis. **a** Drug resistance of HepG2 and fused cells examined with different DOX concentrations (*n* = 4 for three independent experiments. ***P < 0.01, **P < 0.05. Statistical significance was determined using two-tailed Student’s *t*-test. **b** Drug resistance-related gene expressed significantly in fused cells compared with HepG2

Drug resistance of the fused cells was also examined. Fused cells and HepG2 were treated using different concentrations of doxorubicin (DOX) for 48 h. Figure 4a illustrates that the survival rate of the fused cell was significantly higher than that of HepG2 and the survival rates of the fused cell and HepG2 decreased as DOX concentration increased. In the present study, survival rate was defined as the number of viable cells after DOX treatments divided by the number of viable cells without DOX treatments. The results demonstrated that the fused cells exhibited increased drug resistance compared with their donor HepG2 cells.

The qPCR test results indicated that CD133 (Fig. 3b), ALDH1A1, ATP-binding cassette sub-family B member 1 (ABCB1), and B-cell lymphoma 2(Bcl-2) (Fig. 4b) were highly expressed in the fused cells. High expression of CD133 is associated with drug resistance and relapses in a variety of solid tumors. ALDH is an effective detoxifying enzyme, and a high expression of ALDH can provide a route for tumors to resist chemotherapy. The expression of ABCB1 has the capacity of active DNA repair and resistance to apoptosis [24, 25]. The anti-apoptotic protein Bcl-2 has been proven to cause resistance to cancer treatment by forming heterodimers with a number of pro-apoptotic proteins [26, 27]. Bcl-2 also contributes to the resistance to a wide spectrum of chemotherapeutic agents [28, 29]. The expression of CD133, ABCB1, Bcl-2, and ALDH1A1 in the fused cells explains the increased drug resistance of the fused cell [30], suggesting that the cancer cell would acquire drug resistance through cell fusion with the stem cell.

### Fused cells are highly tumorigenic

**Fig. 5:**
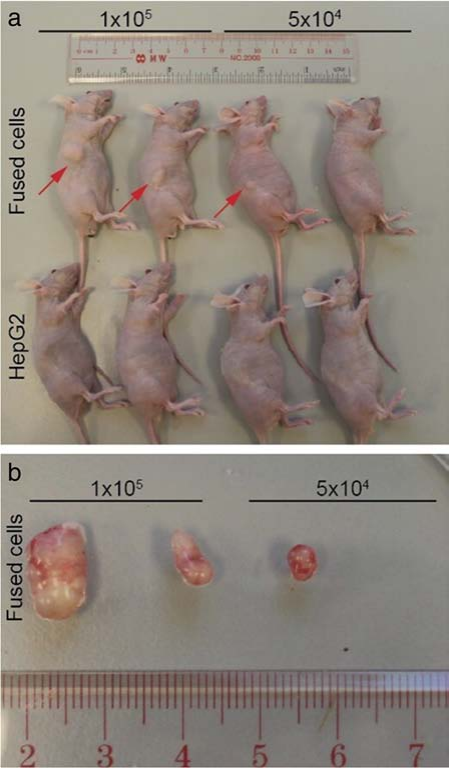
Enhanced tumorigenicity of the fused cells was detected in nude mice. The in vivo test for tumorigenicity of the fused cells. **a** Mice injected with the fused cells (top line) and HepG2 (bottom line) in the number of 5 × 10^4^ and 1 × 10^5^. Red arrows indicate the tumors from the fused cell, whereas in the control group, no tumor was found. **b** Tumors stripped from the corresponding mice

**Fig. 6:**
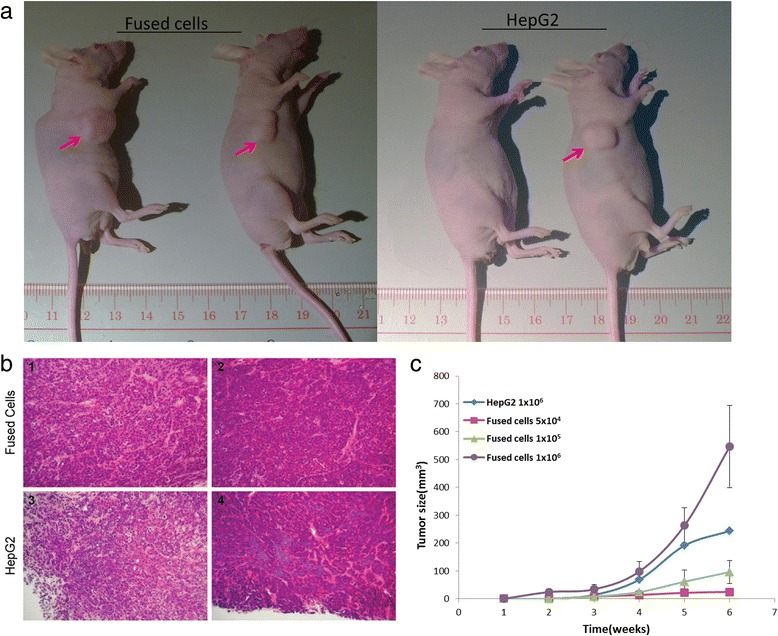
Histological analysis of the in vivo tumor. The tumors were stained with hematoxylin-eosin (H&E). **a** Tumors (arrows) used for the histological studies, both group were injected with 1 × 10^6^ cells. **b** The tumors were stained with H&E. Panels 1, 2 were used to stain tumors induced by 1 × 10^6^ fused cell and panels 3, 4 were employed to stain tumors induced by the 1 × 10^6^ HepG2 cells. **c** Tumor size detected six weeks after cell injection. In the same injection cell number 1 × 10^6^, the tumor caused the faster growth of fused cells than the tumor caused by HepG2

To analyze tumor-initiating capability, nude mice were transplanted with various amounts of the fused cells. The same amounts of HepG2 cells were used as the control. The tumor volume was measured weekly. The fused cells could result in progressively growing tumors in the nude mice with fewer cells compared with HepG2 cells. The injection of fused cells at 5 × 10^4^ facilitated the progressive growth of tumors in the nude mice, whereas the injection of HepG2 cells at 5 × 10^4^ did not induce this effect (Fig. 5). Tumor incidence increased when the injection dose of the fused cells was raised to 1 × 10^5^. However, tumors were still not formed in the mice injected with the same amount of HepG2 cells. When the injected cells were increased to 1 × 10^6^, both the fused and HepG2 cells promoted tumor growth in the nude mice. The fused cells induced 100 % tumor incidence, whereas the HepG2 cells induced only 50 % tumor incidence. The fused cell possessed higher tumorigenicity and faster tumor growth than the HepG2 cells (Table 3 and Fig. 6c).

**Table 3:** In vivo tumor development experiments of HepG2 cells and fused cells in nude mice

Cell type	Cell numbers injected	Tumor incidence^a^	Latency (days)^b^
HepG2	5 × 10^4^	0/4	—
	1 × 10^5^	0/4	—
	1 × 10^6^	1/2	14
Fused cells	5 × 10^4^	2/4	14
	1 × 10^5^	3/4	14
	1 × 10^6^	4/4	12

^a^Number of tumors detected/number of injections

^b^Approximate number of days from tumor cell injection to appearance of a tumor

**Fig. 7:**
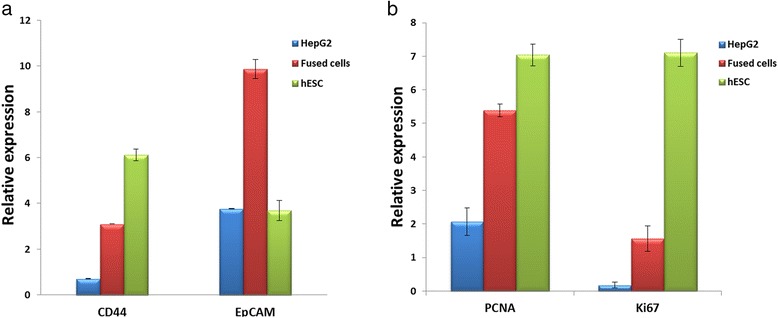
Tumorigenicity-related gene expression analysis. The tumorigenicity-related gene expressions were detected by qPCR. **a** CD44 is a cell surface molecule that affects tumorigenesis. EpCAM is the activator of WNT pathway, which is important in cancer cells. **b** PCNA and Ki67 are tumor proliferation cell nuclear antigens. All of these genes are highly expressed in the fused cells (*p* < 0.05)

As shown in Fig. 5a and b, the xenograft tumors exhibited similar histological features in the mice injected with the fused cells or HepG2 cells, which indicated that the tumor tissue was not a teratoma induced by hESCs. The result of in vivo tumorigenicity assay showed that the fused cells exhibited increased tumorigenicity in vivo. The highly expressed CD133 (Fig. 3), CD44, and EpCAM may illustrate the mechanism of enhanced tumorigenicity (Fig. 7a). The expression of two tumor proliferation factors, proliferating cell nuclear antigen (PCNA) and ki67 were also detected. The qPCR results indicated that both PCNA and Ki67 were expressed relatively high in the fused cell (Fig. 7b). The high expression of PCNA indicates the high risk of carcinogenesis [31]. Ki67 is a nuclear antigen expressed in a variety of solid malignant tumors, and its expression level is significantly higher than that of normal tissue. Ki67 is closely related to the development, metastasis, and prognosis of malignant tumors [32, 33].

## Discussions

Using engineered laser-induced single-cell fusion technology, we have successfully produced a fused cell line, which coalesces the gene expression information of hepatocellular carcinoma cells and stem cells. Experimental results verified that the fused cells are highly tumorigenic and chemoresistant compared with the donor hepatocellular carcinoma cells. The fusion of stem cells and cancer cells enables the hybrids to exhibit stemness and cancer characteristics. Traditionally, during stem cell induced reprograming of somatic cell via fusion, the somatic pluripotency gene epigenetic modifications has been recognized to be a crucial factor, which has been explained as one of the parental cells dominating phenotype over the other [34]. The epigenetic modifications include chromatin remodeling, histone modification and DNA demethylation [35, 36]. However, the mechanism of fusion between cancer cells and stem cells are rarely elaborated.

Our study on artificially engineered fusion of stem cells and liver cancer cells demonstrated a possible mechanism of the tumor-initiating cell generation in the human body. The fusion of stem cells can occur spontaneously in vivo after stem cell injection or transplant [37]; thus, our research may explain the risk induced by stem cell therapy in liver cancer. On one hand, some stem cell therapies may generate cancer stem-like cells and exacerbate cancer instead of curing the disease. On the other hand, the artificial generation of tumor initiating-like cells can be used to produce malignant cells for cancer stem cell drug screening and studies on its mechanism.

## Conclusions

We artificially fused a HepG2-hESC cell pair and isolated a fused cell line. The genetic analysis result demonstrated that the fusion of cancer cells with stem cells resulted in cancer cells that were more tumorigenic and exhibited higher chemoresistance. The result also indicated that the fused cells were highly similar to tumor-initiating cells in biological processes or cellular compartments. The related gene expressions, such as CD133, CD44, and EpCAM, in the fused cells also supported this conclusion. In the future work, we will continue to explore the stem cell mechanism (e.g., functional protein assay and GO comparison to original TICs), as well as conduct more sufficient in vivo experimental study.

## Abbreviations

hESC: human embryonic stem cell
HCC: hepatocellular carcinoma
TSCs: tumor stem cells
TICs: tumor-initiating cell
DOX: doxorubicin
MOI: multiplicity of infection
H&E: hematoxylin-eosin
ALDH1A1: aldehyde dehydrogenase 1 family, member A1
ABCB1: ATP-binding cassette sub-family B member 1
Bcl-2: B-cell lymphoma 2
PCNA: proliferating cell nuclear antigen
